# The immunomodulatory mechanism of acupuncture treatment for ischemic stroke: research progress, prospects, and future direction

**DOI:** 10.3389/fimmu.2024.1319863

**Published:** 2024-05-02

**Authors:** Hongjun Kuang, Xinzhou Zhu, Huan Chen, Han Tang, Hong Zhao

**Affiliations:** ^1^ Department of Acupuncture and Moxibustion, Shanghai University of Traditional Chinese Medicine, Shenzhen Hospital, Shenzhen, China; ^2^ Department of Acupuncture and Moxibustion, Luohu District Hospital of Traditional Chinese Medicine, Shenzhen, China; ^3^ The Brain Cognition and Brain Disease Institute (BCBDI), Shenzhen Institute of Advanced Technology, Chinese Academy of Sciences, Shenzhen, China; ^4^ Shenzhen-Hong Kong Institute of Brain Science-Shenzhen Fundamental Research Institutions, Shenzhen, China; ^5^ Institute of Acupuncture and Moxibustion, China Academy of Chinese Medical Science, Beijing, China

**Keywords:** immunomodulatory mechanism, acupuncture treatment, ischemic stroke, research progress, future direction, prospects

## Abstract

Ischemic stroke (IS) is one of the leading causes of death and disability. Complicated mechanisms are involved in the pathogenesis of IS. Immunomodulatory mechanisms are crucial to IS. Acupuncture is a traditional non-drug treatment that has been extensively used to treat IS. The exploration of neuroimmune modulation will broaden the understanding of the mechanisms underlying acupuncture treatment. This review summarizes the immune response of immune cells, immune cytokines, and immune organs after an IS. The immunomodulatory mechanisms of acupuncture treatment on the central nervous system and peripheral immunity, as well as the factors that influence the effects of acupuncture treatment, were summarized. We suggest prospects and future directions for research on immunomodulatory mechanisms of acupuncture treatment for IS based on current progress, and we hope that these will provide inspiration for researchers. Additionally, acupuncture has shown favorable outcomes in the treatment of immune-based nervous system diseases, generating new directions for research on possible targets and treatments for immune-based nervous system diseases.

## 1 Introduction

Ischemic stroke (IS) is an acute cerebrovascular disease in which blood cannot be supplied to the brain due to a sudden blockage of cerebral vessels (1). IS continues to be the third-leading cause of mortality and disability worldwide, accounting for 5.7% of all deaths and 11.6% of all disabilities worldwide (2). IS survivors often suffer from disabilities that cause a poor quality of life and a heavy economic burden (3). In addition, several comorbidities caused by IS, such as melancholy, epilepsy, exhaustion, and pain, further impair the quality of life of patients (4–7).

An increasing number of studies have shown that the immune response is a double-edged sword in the pathophysiology of IS and has become a breakthrough target in treatment strategies (8). IS induces a series of peripheral and central immune reactions that are crucial to the pathophysiology of development, acute damage cascades, and the chronic course after an IS (9). The acute and chronic phases of IS and the long-term effects of stroke are greatly affected by the immune system (10). The immune response after IS involves innate and adaptive immunity in the central and peripheral nervous systems (11). Targeting regulatory immunity is a promising approach for reducing stroke-related dysfunction.

Acupuncture is a traditional non-pharmacological treatment that dates back thousands of years. It uses needles to penetrate the skin at specific acupoints on the body, head, and ears. Patients with IS receive acupuncture as a complementary medical treatment in various countries, including China, Korea, and the USA (12, 13). Numerous lines of evidence indicated that acupuncture was effective and safe for IS and its complications (14, 15). In addition, acupuncture has been shown to modulate immune responses, particularly neuroimmune responses (16, 17). However, no review has outlined the immunomodulatory mechanisms underlying the effects of acupuncture in the treatment of IS.

In this review, we summarize the immune response following an IS, as well as the immunomodulatory effects of acupuncture in the treatment of IS and its consequences. In addition, we propose some new considerations for the future research direction of acupuncture for IS to provide a new strategy for stroke rehabilitation based on neuroimmune.

## 2 Immune responses after an ischemic stroke

### 2.1 Immune cell response after an ischemic stroke

Immune cells are the primary components of the central immune system. Immune cells play a vital role in regulating the progression of IS. These immune cells include microglia, astrocytes, oligodendrocytes (OLs), neutrophils, T cells, B cells, dendritic cells, and macrophages.

#### 2.1.1 Microglia—the first line of defense

Microglia are frequently found as resident immune cells in the central nervous system (CNS) and affect pathological processes such as neuronal healing, antigen presentation, and poststroke inflammation (18). The functions of microglia and monocytes/macrophages in cleaning debris, triggering neuroinflammation, causing tissue damage, and encouraging tissue recovery are believed to be a double-edged sword (19). After an IS, with the release of damage-associated molecular patterns (DAMPs) and cytokines, the microglia are activated and react. The activation of microglia from the brain and macrophages from the blood composes the first line of defense, which is the innate immune response (20). The two main types of microglia, M1-like and M2-like, can be distinguished based on their biological role and the cytokines and chemokines they secrete (21). Two types of microglia play different roles. Clearing cellular debris, secreting anti-inflammatory substances, promoting angiogenesis, enhancing angiogenesis, and synaptic remodeling are performed by M2-like microglia (22, 23). M2-like microglia inhibited inflammatory damage to protect the blood–brain barrier (BBB) by releasing interleukin (IL)-4, IL-10, and transforming growth factor-β1 (TGF-β1) (24). Ischemic neurons cause M1-like microglia to become polarized, and M1-like microglia release proinflammatory substances that wreak havoc in the nervous system and harm neurons (25). M1-like microglia play a hazardous role after IS, such as damaging the BBB and aggravating the harmful inflammation response (26). Most of the activated microglia in the subacute and chronic phases of stroke are M1-like, which is detrimental to brain regeneration, while M2-like microglia predominate in the first 24 h following stroke (27, 28). Some research has verified that the regulation of microglial polarization can be a promising target to treat IS (29, 30).

#### 2.1.2 Astrocytes—double-edged sword for modulation

The most prevalent glial cell type in the CNS, astrocytes, has an essential function (31). Similar to microglia, damaged brain tissues with the secretion of cytokines and DAMPs stimulated the receptors of astrocytes to differentiate phenotype (32). Astrocytes are actived into two polarization states after an IS, named A1 and A2, facilitating the understanding of the reactive state of astrocytes. Numerous processes are related to astrocytes, including homeostasis, neuronal and synaptic development, control of cerebral blood flow, and vascular remodeling and repair (33, 34). A2-subtype astrocytes secrete substances that facilitate the recovery of neurons, including neurotrophic factors, glutamic acid, homocysteine, and cholesterol (35). Nevertheless, A1-subtype astrocyte development is detrimental to neurons, as it prevents effective axonal regeneration (36). M1-like microglia can promote astrocyte activation (34). More *in vivo* and *in vitro* research should be conducted to explore astrocyte pathways in the case of IS.

#### 2.1.3 Oligodendrocytes—for preserving axonal integrity

OLs, which create myelin, are discovered in white matter (37). When OLs are affected by ischemic damage, OLs produce tissue demyelination and axonal instability, resulting in various neurological deficits (38). Damaged OLs were replaced to restore saltatory conduction and relieve motor function after an IS. Increasing myelin production, augmenting synaptogenesis, promoting oligodendrogenesis, promoting OLs maturation, and suppressing remyelination are the strategies to target OLs for the treatment of IS (39).

#### 2.1.4 Neutrophils—infiltration from blood

Neutrophils are the first immune cells transported to the CNS from the peripheral immune system after stroke (40). The enhancement in neutrophils results in the disruption of cerebral edema, brain injury, and the BBB after an IS (41). Neutrophils also impact thrombosis and atherosclerosis, which are high risk factors of IS (42, 43). Neutrophils invade the brain during the hyperacute, acute, and subacute phases (44). Importantly, circulating neutrophils are related to stroke severity, stroke outcomes, and stroke prognosis (45). The responses of neutrophils after an IS include three parts: (1) neutrophil activation and recruitment, (2) neutrophil adhesion to endothelial cells, and (3) neutrophil transmigration and neurovascular interactions (46). Cytokines, chemokines, and DAMPs act on neutrophils, which cause the recruitment of neutrophils to the site of injury and the delivery of adhesion molecules (47). Neutrophils are key modulators of IS injury. Thus, researchers show a great interest in neutrophils as treatment targets to prevent IS and relieve ischemic brain injury. The processes of neutrophil recruitment and transmigration can also be potential targets to influence stroke severity in detail (48).

#### 2.1.5 T lymphocytes—their conflicting roles in ischemic stroke

T lymphocytes, which are adaptive immune cells, exacerbate ischemic–reperfusion injury in acute IS, in both an antigen-dependent and an antigen-independent manner (49). T lymphocytes release interferon-gamma (IFN-γ), IL-21, and IL-17, which cause harmful effects in IS (50). On the basis of cell functions, T cells are divided into multiple types marked by CD3 expression, including CD8+ cytotoxic T lymphocytes, CD4+ T helper cells, regulatory T cells (Tregs), and γδ T cells (51). CD4+ T cells are the main effector T cells, which modulate brain inflammation through releasing cytokines after an IS (52). CD4+ T cells play a double-edged role in inflammation regulation after a stroke, with anti-inflammatory and proinflammatory effects. CD8+ T cells play a cytotoxic role and promote cell apoptosis by recognizing antigens on T-cell receptors and releasing granzyme and perforin subsequently after an IS (53). Tregs are a type of T cell that influences immune responses after an IS (54). Tregs secrete anti-inflammatory cytokines, inhibit proinflammatory cytokines, induce cell lysis, promote neural regeneration, and modulate microglial and macrophage polarization after an IS (54). It is currently uncertain if Tregs are helpful or harmful in IS, as well as how they affect IS at different phases. Peripheral γδ T cells infiltrated the lesion site after an IS and aggravated BBB injury (55). Numerous γδ T cells infiltrate the ischemic penumbra, and knockout of γδ T cells relieves motor dysfunction and significantly reduces infarction and BBB damage (55). Though the major function of T cells has already been discovered, their specific role and the mechanism of T cells after a stroke need more attention.

In conclusion, the summary of the type and function of immune cells in IS is shown in **Table 1**.

**Table 1:** Summary of type and function of immune cells in ischemic stroke.

Immune cell type	Phenotype	Function
Microglia	M1-like microglia	Damaging the blood–brain barrier, aggravating the harmful inflammation response, and harming neurons
	M2-like microglia	Clearing cellular debris, secreting anti-inflammatory substances, promoting angiogenesis, inhibiting inflammatory damage, and protecting the blood–brain barrier
Astrocytes	A1 subtype astrocytes	Preventing effective axonal regeneration
	A2 subtype astrocytes	Facilitating the recovery of neurons and releasing neurotrophic factors, glutamic acid, homocysteine, and cholesterol
Oligodendrocytes	/	Creating myelin and producing tissue demyelination and axonal instability
Neutrophils	/	Destroying neurons and the blood–brain barrier
T cells	CD4+ T cells	Exerting anti-inflammatory and proinflammatory effects
	CD8+ T cells	Playing a cytotoxic role and promoting cell apoptosis
	Regulatory T cells	Secreting anti-inflammatory cytokines, inhibiting pro-inflammatory cytokines, inducing cell lysis, promoting neural regeneration, and modulating microglial and macrophage polarization
	γδ T cells	Aggravating the blood–brain barrier injury

### 2.2 Immune cytokines’ responses after an ischemic stroke

Immune cytokines secreted by immune cells are promising prognostic and predictive biomarkers for predicting stroke outcomes and therapeutic targets in patients with IS (56). They affect infarct and edema volumes (57). The classification of immune cytokines includes proinflammatory and anti-inflammatory cytokines on the basis of their function.

#### 2.2.1 Proinflammatory cytokines

The proinflammatory cytokines mediate brain tissue destruction after an IS. Tumor necrosis factor (TNF), IL-1β, IL-6, IFN-γ, and IL-17 are classical and well-known proinflammatory cytokines (58). TNF-α is produced by various immune cell types and is related to various physiological events, including endothelial necroptosis, breakdown of the BBB, and the results of stroke (26). After a stroke, the expression of TNF-α, IL-1β, and IL-6 increased, aggravating the brain injury (59). IFN-γ, which is a proinflammatory cytokine, is pivotal for activating microglia into neurotoxic phenotypes that induce oxidative stress, severe dysfunction, and neuron death (60). The level of IL-17 increased in patients with IS. IL-17 accelerates neutrophil recruitment to the ischemic hemisphere (61).

#### 2.2.2 Anti-inflammatory cytokines

IL-10, TGF-β1, and IL-4 are classical anti-inflammatory cytokines that mediate neuroprotection. IL-10 has a role in clear vascular endothelial protective properties and neuronal protection after brain injury (62). Once the level of TGF-β1 was upregulated, the apoptosis of neurons was suppressed (63). IL-4 modulates the proliferation of B cells and T cells, and the differentiation of B cells. Enhancing signaling through the IL-4 receptor may alleviate inflammation after ischemia and attenuate sensorimotor and cognitive deficits (64).

#### 2.2.3 Multiple effects cytokines

The current study considers that IL-21 is an anti-inflammatory cytokine after an IS. IL-21R-deficient mice had reduced collateral vascular connections and increased brain infarct volume after ischemic brain injury. Moreover, the IL-21 receptor exerts neuroprotective effects in IS mice (65). However, IL-21 promotes the proinflammatory effects of macrophages during respiratory infection (66).

### 2.3 Immune organ responses after an ischemic stroke

Immune-related organs include the spleen, bone marrow, thymus, lymph nodes, lungs, liver, adrenal glands, and gut (67). The bone marrow exudes plenty of proinflammatory cells and cytokines, resulting in exacerbating peripheral inflammation (68). The gut changes intestinal dysbiosis to affect peripheral inflammation via regulating the gut–brain axis (69). The liver metabolism takes part in immunosuppression, inflammation, and oxidative stress after an IS (70). IS causes pulmonary damage, harmful inflammation, and reduction of alveolar macrophage phagocytic capability, which is related to the brain–lung axis (71). Most studies focus on the changes in the spleen and thymus after an IS, but the research depth is insufficient. Additionally, the function and change of bone marrow, lymph glands, lungs, and intestines after an IS should receive more attention.

#### 2.3.1 The spleen

Spleen weight and size dramatically decreased in middle cerebral artery occlusion (MCAO) rats and mice, and spleen atrophy and cell death occurred after ischemia (72–74). The number of serum leukocytes is adversely linked to changes in spleen size in patients (75). At 24 h after ischemia, MCAO rats with splenectomies had lower levels of proinflammatory cytokines, fewer T cells, neutrophils, and macrophages in the brain tissue, and higher levels of anti-inflammatory factor IL-10, resulting in a smaller infarct size in the brain (76).

#### 2.3.2 The thymus

At 3, 7, and 13 days after reperfusion, the number of CD8α^+^ T cells in the spleen and thymus of 90-min ischemia–reperfusion (I/R) rats decreased significantly, the TUNEL^+^ apoptotic cells in the spleen increased significantly, and the numbers of Iba1^+^ macrophages, CD68^+^ macrophages, and Ki67^+^ proliferating cells decreased significantly in the spleen and thymus of 90-min I/R rats and increased in brain tissue (77). The number of CD3+ T cells in the spleen and thymus of 30-min I/R mice was enhanced 24 h after stroke (78). The number of microglia, the level of TNF-α, and the inflammatory microbiota in the spleen and thymus of 60-min I/R rats increased significantly (79).

## 3 The mechanism of acupuncture treatment regulating immunity in ischemic stroke

In this review, acupuncture treatment includes electroacupuncture (EA) and manual acupuncture (MA). Acupuncture treatment stimulates acupoints to modulate the neuroendocrine–immune network (80). When dealing with various pathological conditions, acupuncture treatment can modulate immune system function, immune cell motivation and phenotypic metamorphosis, and immune cytokine expression. In this section, the mechanism of acupuncture treatment on immunity after an IS was extracted and summarized. As shown in **Tables 2**, **3**, authors, animal model, acupoint selection, acupuncture method, stimulation parameter, treatment course, and neuroimmune molecular and cellular results were extracted and summarized (**Tables 2**, **3**).

**Table 2:** Summary of studies exploring the effects of acupuncture treatment on ischemic stroke by modulating the central nervous system.

Author	Animal model	Acupoint	Acupuncture method	Stimulation parameter	Treatment course	Neuro-immune molecular and cellular results
Han B 2015	MCAO male Wistar model rats	Neiguan (PC6), Quchi (LI11), Diji (SP8)	EA	2/15 Hz, 1 mA, 1.5 seconds	30 min/days, for consecutive 5 days.	Downgrade: TLR4, HMGB1, TRAF6, IKKβ, NF-κB p65 in microglia; TNF-α, IL-1β, IL-6 in brian tissues
Cao Y 2020	CCH male Wistar model rats	Zusanli (ST36), Baihui (GV20)	MA	Twirling reinforcing manipulation	once daily for 2 weeks (continued for 6 days and 1 day of rest) from the third day after surgery	Downgrade: Iba-1, TNF-α, IL-6 in the hippocampus Upgrade: α7nAChR in neurons, JAK2, STAT3 in Hippocampus
Yang J 2019	CMi male Wistar model rats	Zusanli (ST36)	MA	Twirling reinforcing manipulation	once daily for 2 weeks (with a rest every seventh day)	Downgrade: NF-κB p65 nuclear translocation, hydroxyl radical generation, Ca2+ homeostasis
Jittiwat J 2019	pMCAO male Wistar model rats	Baihui (GV20)	LA	a laser spot diameter of 100 μm, with a laser module output of 100 mW	Once daily for 14 days	Downgrade: IL-6 in the hippocampus Upgrade: GSH-Px, SOD
Li Z 2020	pMCAO male SD model rats	Baihui (GV20)	MA	The needle was rotated for 1 min at a frequency of 200 rpm and twirled once an hour for 2 hours.	Once daily for three weeks	Downgrade: TNF-α, IL-1β, ROS in the hippocampus Upgrade: BDNF, S100b, GFAP in the hippocampus
Xu H 2014	2h CIRI male SD model rats	Baihui (GV20), Zusanli (ST36)	EA	continuous-wave, 2Hz, 1mA	once a day for 6 days	Downgrade: TNF-α, HSP70 in serum
Xu H 2014②	2h CIRI male SD model rats	Baihui (GV20), Zusanli (ST36)	MA; EA	MA: twisted 180 degrees at a rate of 10065 twists per min for 1 min, with the twisting procedure repeated at 10-min intervals; EA: continuous-wave, 2Hz, 1mA	Twice befor euthanasia	Downgrade: MMP2, AQP4, AQP9, MPO+ and CD68+ cell in the ischemic penumbra and the core zone
Liu R 2020	pMCAO male SD model rats	Baihui (GV20), Dazhui (GV24)	EA	sparse-dense, 20Hz, 1-2mA, 30min	Once per day for three days	Downgrade: TNF-α, IL-1β, NF-κB, Iba-1, CD11b
Lan L 2013	2h I/R male SD model rats	Quchi (LI11), Zusanli (ST36)	EA	1/20Hz; an intensity of the muscle twitch threshold	30min, twice	Downgrade: TLR4, NF-κB p65, IκB, TNF-α, IL-1β, IL-6
Wang W 2016	2h I/R male SD model rats	Baihui (GV20), Shuigou (GV26)	EA	2/150Hz; 3mA	30min, once every 12h, for 3 consecutive days	Upgrade: TGF-β1
Zhou X 2020	2h I/R male SD model rats	Baihui (GV20), Hegu (LI4), Taichong (LR3)	EA	20Hz for 5min and 2Hz for 30min; 1mA	35min; once a day for 72h	Upgrade: ABIN1 in the peri-infarct cortex, Downgrade: NF-κB, TNF-α, IL-1β, MCP-1 in the peri-infarct cortex
Ma Z 2019	pMCAO male SD model rats	Baihui (GV20)	EA pretreatment	dense-disperse frequency of 2/15 Hz; 1mA;	30 min; once per day for 5 days before operation	Upgrade: α7nAChR, Arg-1, TGF-β1, IL-10 in the ischemic penumbra Downgrade: iNOS, IL-1β, TNF-α in the ischemic penumbra
Lin X 2021	2h I/R male SD model rats	Baihui (GV20), Hegu (LI4), Taichong (LR3)	EA	20Hz for 5min and 2Hz for 30min; 1mA	35min; once a day for 72h	Upgrade: ym1, arg1, fizz, CD206+/Iba+ cell, CX3CL1+/CX3CR1+ cell, CX3CL1, Downgrade: iNOS, IL-1β, TNF-α, iNOS+/Iba+cell, NLRP3, CX3CR1
Tao J 2015	2h I/R male SD model rats	Zusanli (ST36), Quchi (LI11)	EA	a dense disperse wave of 1 or 20 Hz	30min; once a day for 3 days	Upgrade: GFAP/Vimentin, GFAP/Nestin, the co-expression of GFAP and BrdU, Cyclin D1, CDK4, p-Rb in peri-infarct cortex and striatum
Xiao Y 2013	MCAO male SD model rats	Zusanli (ST36), Quchi (LI11)	EA	15, 30, 100Hz	30 min; once a day for 5 days	maintain the structural integrity
Liu J 2017	2h I/R male SD model rats	Baihui (DU20), Shenting (DU24)	EA	disperse-dense waves of 2-10 Hz; 2-4 mA	30min; once a day for 7 days, start 2 days after ischemia	Upgrade: α7nAChR in the hippocampus Downgrade: GFAP, Iba1, TNF-α, IL-1β
Yang J 2021	1h I/R male C57BL/6J model mice	Baihui (GV20)	EA pretreatment	2/15Hz; 1 mA	30min; once a day for 5 days	Upgrade: ambient endocannabinoid, astroglial cannabinoid type 1 receptors
Lu Y 2015	MCAO male Wistar model rats	Neiguan (PC6), Quchi (LI11)	EA	2/15Hz; 1 mA	20min; once daily for 7 days	Upgrade: GFAP, MCT1 in the ischemic brain tissue
Luo Y 2011	MCAO male Wistar model rats	Baihui (GV20), Dazhui (GV14)	EA	4/20Hz; 1-3 mA	30min; once a day for 1, 3, 7, and 21 days each group	Upgrade: EAAT2, CX43 Downgrade: GFAP, Ca^2+^ in Astrocytes
Wang SJ 2003	90min I/R male Wistar model rats	D group: Baihui (GV20), Shuigou (GV26); G group: Hanyan (GB4), Xuanlu (GB5), Xuanli (GB6), Qubin (GB7)	EA	3/20 Hz; 3 mA	30min; once every other day	Upgrade: VEGF in astrocytes
Han X 2010	MCAO male Wistar model rats	Baihui (GV20), Dazhui (GV14)	EA	20 Hz; 1-2 mA	30min; once a day for 28 days	Upgrade: GFAP-immunoreactive cells in the peri-infarct region
Ahn SM 2016	BCAS male C57BL/6 model mice	Baihui (GV20), Dazhui (GV14)	EA	2 Hz; 2 V	20min; once daily for 7 consecutive days	Downgrade: neural/glial antigen 2 (NG2) and platelet-derived growth factor receptor-α (PDGFRα) Upgrade: 2,3-cyclic nucleotide-3-phosphodiesterase (CNPase), NT4/5, TrkB, CREB phosphorylation
Lee HJ 2022	40min I/R male C57BL/6 model mice	Sishencong (EX-HN1), Baihui (GV20)	EA	2 Hz; 1 mA	20min; once every other day	Upgrade: NG2-expressing cells, ERK, Akt, BDNF, TGFβ, NT3, pTrkB
Liu W 2016	2h I/R male SD model rats	Quchi (LI11), Zusanli (ST36)	EA	1-20 Hz; 0.2 mA	30min; once a day for three days	Downgrade: Iba-1, ED1 positive microglia, NF-κB p65, IκB-α, p38 MAPK, MyD88 in the peri-infract sensorimotor cortex, TNF-α, IL-1β, IL-6 in serum
Zou J 2021	60min I/R male C57BL/6 model mice	Shuigou (GV26), Chengjiang (CV24)	EA	4/16 Hz; initial voltage 1V with a 1V increase every 10min; terminal tension 3V	30min; both pre- and post-MCAO	Upgrade: ANXA1, Arg-1, BDNF Downgrade: IL-1β, iNOS, TNF-α
Jiang J 2017	2h I/R male SD model rats	Baihui (GV20), Hegu (LI4), Taichong (LR3)	EA	20 Hz and 2 Hz	35min; once a day for 72h	Upgrade: CYLD Downgrade: NF-κB p65, TNF-α, IL-1β, neuronal CX3CL1
Xu H 2018	2h I/R male SD model rats	Baihui (GV20), Hegu (LI4), Taichong (LR3)	EA	2/20 Hz; 1 mA	30min; once a day for 72h	Upgrade: TREM2, p-PI3K/PI3K, p-Akt/Akt, p-NF-kB/NF-kB, Arg-1, IL-10 in the ischemic penumbra Downgrade: TNF-α, IL-1β, IL-6 in the ischemic penumbra
Chen S 2023	MCAO male SD model rats	Shuigou (GV26)	EA	4/20 Hz; 1-3 mA;	30min; twice a day for 24h	Downgrade: Iba-1, IL-1β, IL-6, Lnc826
Wang F 2013	2h I/R male SD model rats	Baihui (GV20)	EA pretreatment	2/15 Hz; 1 mA	30min; once a day for 5 days	Downgrade: NDRG2, TUNEL+
Jin Z 2013	90min I/R MCPIP1 knockout and C57/BL6 model mice	Baihui (GV20)	EA pretreatment	2/15 Hz; 1 mA	30min; once a day for 2 days	Upgrade: monocyte chemotactic protein-induced protein 1 Downgrade: TNF-α, IL-1β, IL-6, MCP-1, NF-κB
Lin W 2016	2h I/R male SD model rats	Zusanli (ST36), Quchi (LI11)	EA	1/20 Hz;	30min; once a day for 72h	Upgrade: miR-9, IκBα Downgrade: NF-κB p65, TNF-α, IL-1β
Wang Q 2012	2h I/R male SD model rats	Baihui (GV20)	EA	2/15 Hz; 1 mA	30min; once a day for 5 days	Upgrade: α7nAChR in ischemic penumbra Downgrade: HMGB1

MCAO, middle cerebral artery occlusion; SD, Sprague–Dawley; EA, electroacupuncture; TLR4, toll-like receptor 4; HMGB1, high mobility group box 1; TRAF6, TNF receptor-associated factor 6; IKKβ, inhibitor of kappa B kinase beta; NF-κB, nuclear factor kappa-B; TNF-α, tumor necrosis factor alpha; IL-1β, interleukin-1beta; Iba-1, ionized calcium binding adapter molecule-1; α7nAChR, α7 nicotinic acetylcholine receptor; JAK2, janus kinase 2; STAT3, signal transducer and activator of transcription 3; CCH, chronic cerebral hypoperfusion; CMi, cerebral multi-infarction; GSH-Px, glutathione peroxidase; SOD, superoxide dismutase; MA, manual acupuncture; ROS, reactive oxygen species; BDNF, brain-derived neurotrophic factor; S100B, S100 calcium binding protein B; GFAP, glial fibrillary acidic protein; HSP70, 70-kDa heat shock proteins; CIRI, cerebral ischemia–reperfusion injury; MMP2, matrix metalloproteinase 2; AQP4, aquaporin protein-4; IκB, inhibitor of NF-κB; ABIN1, A20-binding inhibitor of NF-kappaB 1; MCP-1, monocyte chemotactic protein-1; I/R, ischemia–reperfusion; Arg-1, arginase-1; TGF-β1, transforming growth factor-β1; iNOS, inducible nitric oxide synthase; CX3CL1, CX3C motif chemokine ligand 1; CX3CR1, CX3C motif chemokine receptor 1; NLRP3, NOD-like receptor thermal protein domain associated protein 3; MCT1, monocarboxylate transporter 1; EAAT2, excitatory amino acid transporter 2; CX43, connexin 43; VEGF, vascular endothelial growth factor; NG2, neural/glial antigen 2; PDGFRα, platelet-derived growth factor receptor-α; CNPase, 2,3-cyclic nucleotide-3-phosphodiesterase; ERK, extracellular regulating kinase; TrkB, tyrosine kinase receptor B; MAPK, mitogen-activated protein kinase; MyD88, myeloid differentiation primary response gene 88; ANXA1, annexin A1; CYLD, cylindromatosis; TREM2, triggering receptor expressed on myeloid cells-2; NDRG2, n-myc downstream-regulated gene 2; LA, laser acupuncture; MCT1, monocarboxylate transporter 1; VEGF, vascular endothelial growth factor; NDRG2, N-Myc downstream-regulated gene 2; BCAS, bilateral common carotid artery stenosis.

**Table 3:** Summary of studies exploring the effects of acupuncture treatment on ischemic stroke by modulating peripheral immunity.

Author	Animal model	Acupoint	Acupuncture method	Stimulation parameter	Treatment course	Neuro-immune molecular and cellular results
Deng P 2022	MCAO male C57BL/6 model mice	Baihui (GV20), Zusanli (ST36)	EA	2Hz; 1mA	30min; Once daily for 48h	Downgrade: IFNγ+ Th cells, IL-17+ Th cells, IL-33+ cells, ST2+ cells
Wang YL 2023	2h I/R male SD model rats	Baihui (GV20)	EA	2/15Hz; 1mA	20min, 2h and 24h after ischemia	Downgrade: TNF-α and IL-1β in the brain, serum, and small intestine, CXCL1, CXCL2 expression in small intestine, the percentage of the CD4+ cells, TCRγδ+ cells, γδT cells in small intestine, IL-17A in the peri-ischemic cortex and small intestine Upgrade: IL-10 in serum and small intestine, Tregs percentage, Foxp3+ cells %, and the ratio of Treg to γδT cells in small intestine
Wang Y 2023	2h I/R male SD model rats	Baihui (GV20)	EA	2/15Hz; 1mA	20min, once daily for three days	Downgrade: TNF-α, IL-1β, in the peri-ischemic cortex, ischemic hemisphere, and small intestine; Cxcl1, Cxcl2 in ischemic hemisphere, IL-17A in the peri-ischemic cortex and small intestine, TCRγδ+ cells in ischemic hemisphere and small intestine Upgrade: IL-10 in the peri-ischemic cortex and small intestine, Foxp3+ cells %, The ratio of Treg/γδ T cells in ischemic hemisphere and small intestine
Qin L 2022	severe stroke patients	Old Ten Needles	MA	/	once daily for seven days	Upgrade: albumin, prealbumin, hemoglobin, total lympho-cyte count in peripheral blood
Jin L 2021	Cerebral ischemic stroke patients	Baihui (GV20), Shenting (GV24), Sishencong (EX-HN1), Sanyinjiao (SP6), Zusanli (ST36), Neiguan (PC6), Shenmen (HT7)	MA	/	30min, once daily, for 14 days	Downgrade: CD8+ cell %, CRP, TNF-α, IL-6 in peripheral blood Upgrade: CD3^+^ cell %, CD4^+^ cell %, CD4+ cell %/CD8+ cell % in peripheral blood

IFN-γ, interferon-gamma; TH cell, helper T cell; TCR, T-cell receptor; Tregs, regulatory cells; CRP, C-reactive protein.

### 3.1 The immunomodulatory effect of acupuncture treatment on the central nervous system

#### 3.1.1 The immunomodulatory effect of acupuncture treatment on immunocytes

##### 3.1.1.1 Microglia

Acupuncture improves stroke by modulating microglial polarization and morphology. Moreover, acupuncture treatment regulates microglial markers, receptors, and signaling pathways. A study showed that EA at GV26 and CV24 relieved neurological deficits by enhancing the expression of annexin A1 and its receptor formyl peptide receptor, promoting M2-like microglia polarization and the levels of arginase-1 (Arg-1) and brain-derived neurotrophic factor (BDNF), and reducing the levels of IL-1β, inducible nitric oxide synthase (iNOS), and TNF-α in the cerebral cortex (81). The nuclear factor kappa-B (NF-κB) is a key nuclear transcription factor, which plays a crucial role in the inflammation and immune responses of cells (82). EA at GV20 and GV24 regulated brain injury-induced inflammation by inhibiting NF-κB activation and decreasing the expression of ionized calcium binding adapter molecule-1 (Iba-1), CD11b, TNF-α, and IL-1β, indicating that EA suppressed the activation of microglia and the inflammatory response in brain tissues (83). EA at LI11 and ST36 reduced infarct volume by attenuating the overactivation of Iba-1 and ED1-positive microglia in the peri-infract sensorimotor cortex, decreasing the level of TNF-α, IL-1β, and IL-6 in serum, and preventing the nucleus translocation of NF-κB p65 and the expression of p38 mitogen-activated protein kinase (p38 MAPK) and myeloid differentiation primary response gene 88 (MyD88) in the peri-infract sensorimotor cortex. These outcomes indicated that EA recovered motor impairment by inhibiting microglia-mediated neuroinflammation (84). EA at PC6, LI11, and SP8 in MCAO rats suppressed the toll-like receptor 4/NF-κB (TLR4/NF-κB) signaling pathway to alleviate microglia activation and inflammation in brain tissues (85). EA at GV20, LI4, and LR3 inhibits neuroinflammatory damage by increasing the A20-binding inhibitor of NF-kappaB 1 (ABIN1) and suppressing NF-κB activation in the peri-infarct cortex, mediating the modulation of microglial polarization (86). EA activated the microglia-specific receptor triggering receptor expressed in myeloid cells 2 (TREM2) through PI3K/AKT and NF-κB signaling pathways to decrease inflammation in the CNS (87).

The cholinergic-anti-inflammatory pathway is the efferent arm of the inflammatory reflex, which mediates the prevention of systemic inflammation by stimulating the vagus nerve (88). The cholinergic anti-inflammatory system involves neuroimmune interactions that produce systemic and anti-inflammatory effects through the α7 nicotinic acetylcholine receptor (α7nAChR). EA pretreatment reduced infarct volume and improved neurological deficits by activating the α7nAChR-mediated phenotypic conversion of microglia, thus reducing the inflammatory response in the ischemic penumbra (89). MA at ST36 and GV20 promoted cognitive function and protected neurons in CCH rats via downregulating the expression of TNF-α and IL-6 and reducing the number of microglia in the hippocampus by activating the janus kinase 2/signal transducer and activator of transcription 3 (JAK2/STAT3) pathway by targeting α7nAChR (90). EA pretreatment inhibits high mobility group box 1 (HMGB1) release by α7nAChR activation in I/R rats (91).

Crosstalk between microglia and neurons is a potential target for neuroimmune regulation (92). EA at GV20, LI4, and LR3 plays anti-inflammatory and neuroprotective roles by upregulating cylindromatosis (CYLD) and CX3C motif chemokine ligand 1 (CX3CL1) expression and downregulating CX3C motif chemokine receptor 1 (CX3CR1) expression, suggesting that the crosstalk between microglia and neurons is regulated. Therefore, M1-like microglial polarization is suppressed, and M2-like microglia polarization is prompted (93). Jiang et al. found that EA at GV20, LI4, and LR3 relieved neurological deficits by upregulating CYLD in neurons, preventing the nucleus translocation of NF-κB p65 in neurons and microglia polarization, and downregulating the expression of TNF-α, IL-1β, and neuronal CX3CL1 in the peri-ischemic area (94). EA at GV26 modulates microglial polarization through the Lnc826-mediated hippo/YAP pathway in the cortex (95).

**Figure 1:**
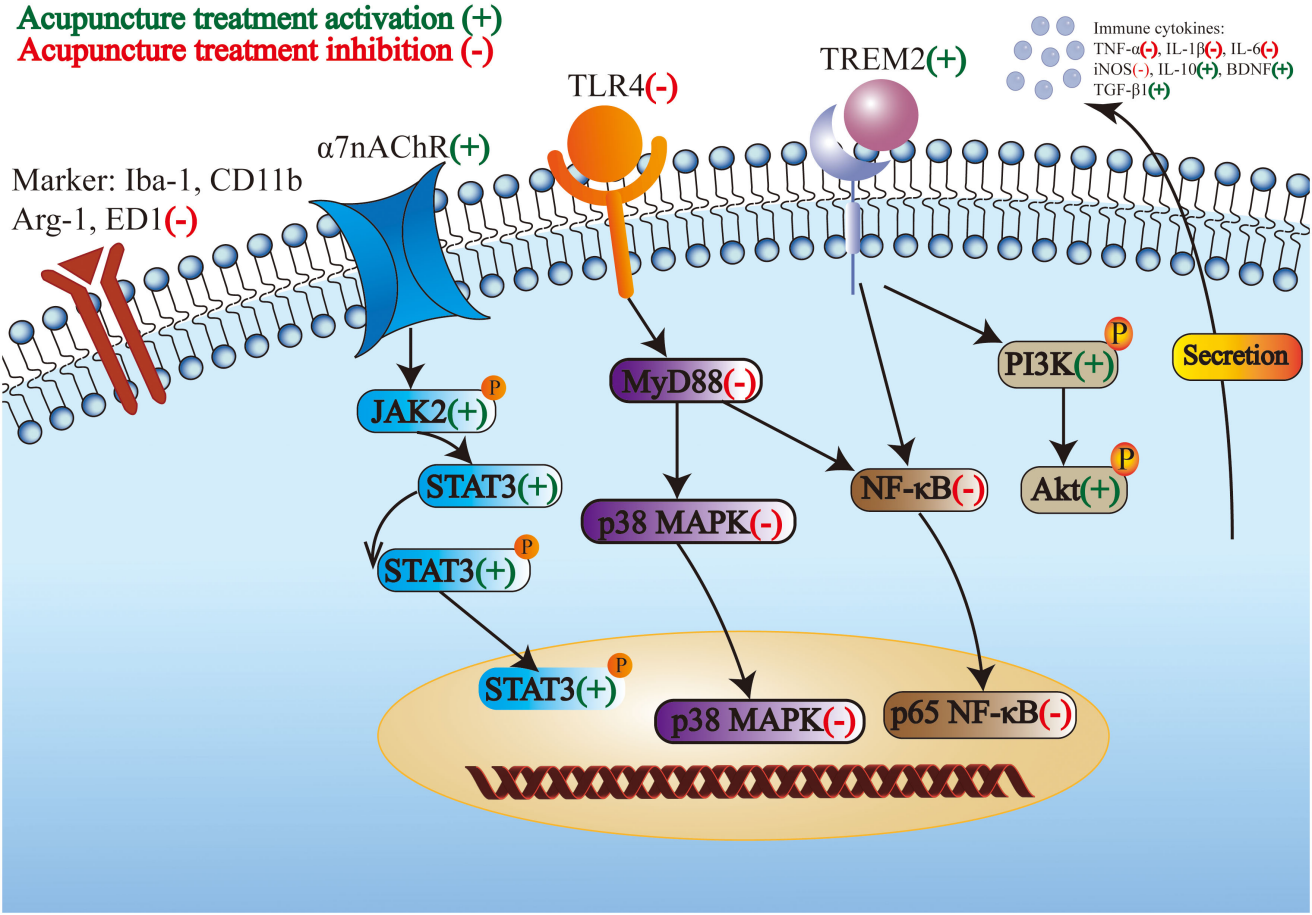
The regulation of microglia in the central nervous system by acupuncture treatment for the treatment of ischemic stroke through multiple targets.

Above all, M1-like microglia is suppressed, M2-like microglia is prompted, and the crosstalk between microglia and neurons is regulated by acupuncture treatment stimulation. In addition, acupuncture treatment inhibits the proinflammatory pathway and activates the anti-inflammatory pathway to modulate the phenotype and morphology of microglia and microglial markers and receptors. Subsequently, the proinflammatory cytokines secreted by microglia decreased and the anti-inflammatory cytokines secreted by microglia increased. The regulation of microglia in the CNS by acupuncture treatment for the treatment of IS through multiple targets is shown in **Figure 1**.

##### 3.1.1.2 Astrocytes

EA improves stroke by regulating the proliferation of astrocytes and the cytokines secreted by astrocytes. EA at ST36 and LI11 maintains the structural integrity of astrocytes in brain tissue (96). Owing to the special double-edged sword function of astrocytes, two opposing results were observed. EA at GV20 and GV14 recovered post-ischemic behavioral dysfunction by activating astrocytes and preventing excess reactive gliosis (97). EA at ST36 and LI11 exerted neuroprotective effects by enhancing the proliferation of astrocytes and increasing the secretion of BDNF from reactive astrocytes in the peri-infarct cortex and striatum (98). Therefore, more research should be conducted to explore the conditions required for astrocyte activation or inhibition. EA at DU20 and DU24 reduced the neuroinflammatory response by activating α7nAChR and decreasing the number of astrocytes (99). EA pretreatment at GV20 upregulated ambient endocannabinoid (eCB) expression and activated ischemic penumbral astroglial cannabinoid type 1 receptors (CB1R) in the ischemic penumbra, protecting neurons from ischemia (100). EA at PC6 and LI11 activated lactate metabolism in resident astrocytes for the use of injured neurons around the ischemic area by enhancing lactate transporter (monocarboxylate transporter 1, MCT1) expression (101). EA at GV20 and GV14 regulated the activation of astrocytes, increased the expression of excitatory amino acid transporter-2 (EAAT2) and connexin 43 (CX43), decreased Ca2+ in astrocytes, and promoted beneficial interactions between astrocytes and synapses in brain tissues (102). EA at GB4, GB5, GB6, and GB7 enhanced the induction of vascular endothelial growth factor in astrocytes of the peri-infarct area after reperfusion (103). EA pretreatment at GV20 attenuated the expression and cellular translocation of the N-Myc downstream-regulated gene 2 (NDRG2) in astrocytes in the ischemic penumbra (104).

In summary, the proliferation and structural integrity of astrocytes were enhanced, excess reactive gliosis is prevented, and the release of beneficial neurotransmitters secreted by astrocytes increased through acupuncture treatment stimulation. Receptors and pathways in astrocytes play an important anti-inflammatory role in these effects.

##### 3.1.1.3 Neutrophils, monocytes/macrophages, and oligodendrocytes

The following studies focused on the regulation of neutrophils, monocytes, macrophages, and OLs. EA and MA at GV20 and ST36 exerted neuroprotective actions in MCAO rats by decreasing the number of immune cells, including neutrophils and monocytes/macrophages (105). EA pretreatment at GV20 reduced inflammatory cytokine production and leukocyte infiltration by inducing the expression of monocyte chemotactic protein-induced protein 1 (MCPIP1) in monocytes in brain tissues (106).

EA at EX-HN1 increased the percentage of CC1-positive cells, indicating that EA promotes the survival and differentiation of OLs (107). EA at GV20 and GV14 ameliorated memory impairment by strengthening OL differentiation from OL precursor cells and by upregulating the Neurotrophin 4/5-tyrosine kinase receptor B (NT4/5-TrkB) pathway in OLs (108).

Few studies focused on the effects of neutrophils, monocytes, macrophages, and OLs after an IS stimulated by acupuncture treatment. These immune cells were believed to be promising targets for the treatment of IS. Hence, these immune cells deserve further study in the future.

#### 3.1.2 Acupuncture treatment effects on immune cytokines

Studies have shown that cytokines are the core mediators of neuroimmune regulatory mechanisms (109, 110). Acupuncture bidirectionally modulates the release of immune cytokines, forming a complex network with multiple effects on IS. Acupuncture regulates immune cytokines from immune cells to control multiple immune responses after an IS. The scalp acupuncture at GV20 was determined to counteract ischemic brain injury through downregulation of TNF-α and IL-1b in the hippocampi (111). EA at GV20 and ST36 exerted neuroprotective properties in CIRI rats by reducing TNF-α and 70-kDa heat shock proteins (HSP70) (112). EA at GV20 and GV26 improved neurological function by increasing the serum level of TGF-β1 (113). LA at GV20 alleviated cognitive and motor deficits by reducing the expression of IL-6 and improving glutathione peroxidase (GSH-Px) and superoxide dismutase (SOD) activity in the hippocampus (114).

The NF-κB signaling pathway is a well-known nuclear transcription factor and the most studied proinflammatory biomarker and target of IS. MA at ST36 protected cognitive function in cerebral multi-infarction rats by suppressing NF-κB p65 nuclear translocation in hippocampal tissues (115). EA at ST36 and LI11 prevented neuroinflammation by regulating the miR-9-mediated NF-κB signaling pathway in the ischemic cortex (116). EA at LI11 and ST36 on the paralyzed limb showed neuroprotective activity by exhibiting the TLR4/NF-κB pathway (117).

Hence, proinflammatory cytokines were reduced, anti-inflammatory cytokines were upregulated, and the proinflammatory signaling pathway was suppressed by acupuncture treatment stimulation.

### 3.2 The immunomodulatory effect of acupuncture treatment on peripheral immunity

Acupuncture treatment regulates peripheral immunity, especially immune cells in the peripheral blood and immune cells in the spleen. EA at GV20 and ST36 improved motor function and brain damage in MCAO rats by decreasing IFNγ+ Th cells and IL-17+ Th cells in spleen tissues and peripheral blood (118). EA at GV20 alleviated cerebral injury by reducing the level of TNF-α and IL-1β in the brain, serum, and small intestine; reducing IL-17A, CXCL1, and CXCL2 expression, and the percentage of CD4^+^ cells, TCRγδ^+^ cells, and γδT cells in the small intestine; increasing the level of IL-10 in the serum and small intestine; and increasing Tregs percentage and the ratio of Treg to γδT cells in the small intestine (119, 120). Those studies indicated that EA regulated the differentiation of T-cell subsets in the small intestine and promoted the balance of Treg/γδ T cells toward Tregs. MA increased the total lymphocyte count in IS patient’s peripheral blood (121). MA increased IS patient’s CD8+ cell percentage and the level of CRP, TNF-α, and IL-6 in peripheral blood, and decreased CD3^+^ cell percentage, CD4^+^ cell percentage, the ratio of CD4^+^ cell percentage, and CD8+ cell percentage in peripheral blood (122). Therefore, major studies focus on suppressing harmful T cells in spleen tissues and peripheral blood, decreasing the expression of proinflammatory cytokines in serum and the small intestine, increasing the expression of anti-inflammatory cytokines in serum and the small intestine, and increasing the beneficial T cells in peripheral blood.

**Figure 2:**
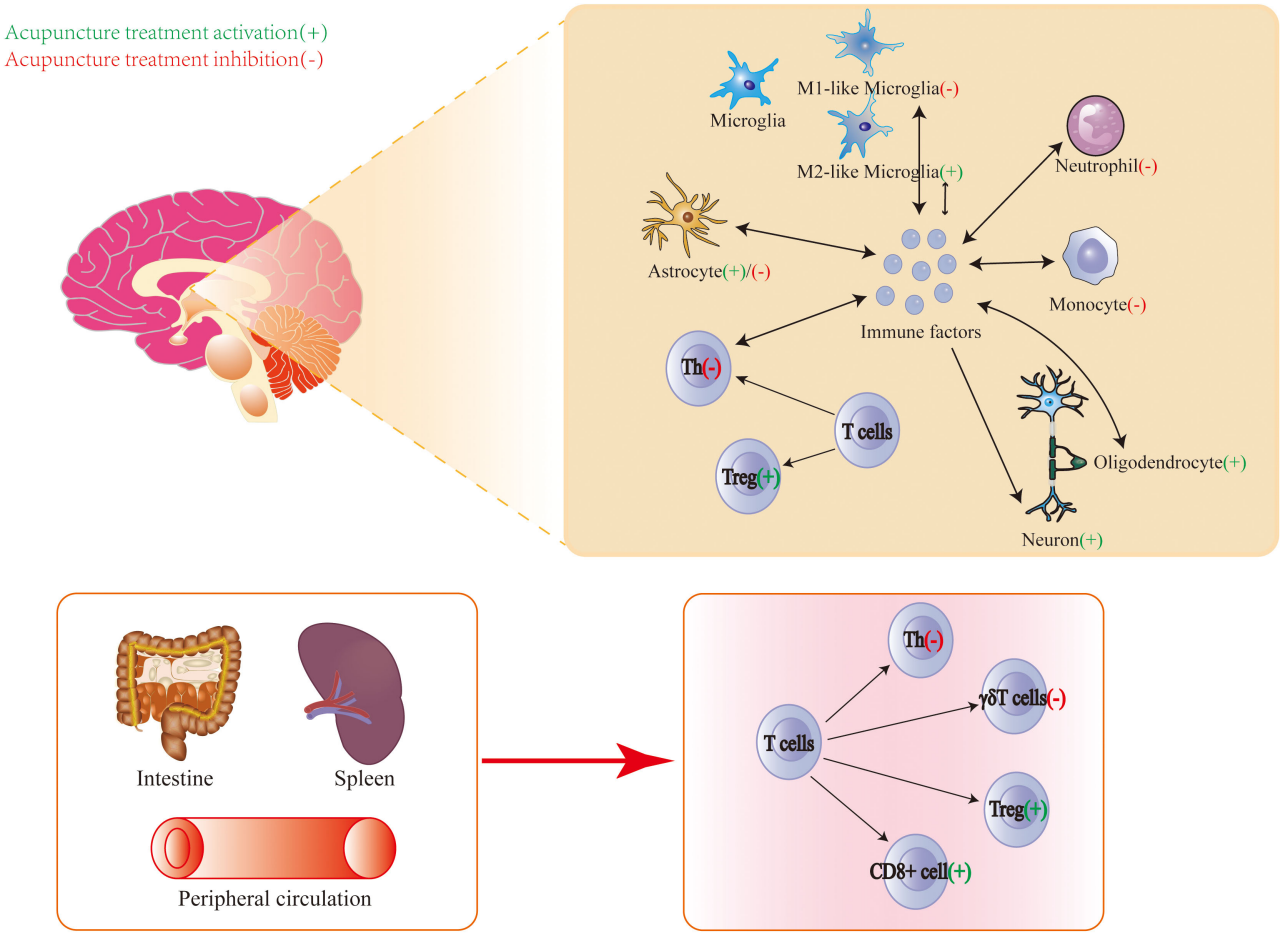
Central and peripheral immune modulation by acupuncture treatment for the treatment of ischemic stroke.

Above all, the central and peripheral immune modulation by acupuncture treatment for the treatment of IS is shown in **Figure 2**.

## 4 Factors influencing acupuncture treatment effect

The operation time of acupuncture is an important factor that influences the efficacy of acupuncture treatment. Gao et al. found that for stroke patients, acupuncture was more effective in the morning than in the afternoon (123). The effect of EA at GV20 and GV14 in the subacute phase was better than that of EA in the acute phase (124). Needle retention time had a significant effect on the efficacy of acupuncture treatment. He et al. considered the time–effect relationship between needle retention time and acupuncture efficacy for patients with IS (125). The efficacy of the 60-min needle retention time was better than that of the 40-min and 20-min needle retention times (125). A similar conclusion was reached in MCAO rats. When comparing neural function recovery, neurobehavioral scores were greater in the 48-h group than in the 12-h group and in the 24-h group (126). The output frequency of the EA instrument affects its efficacy. The efficacy of EA at 15 and 30 Hz was better than the efficacy of EA at 100 Hz in MCAO rats (96). The perceived stimulation intensity of MA had a differential effect on cerebral activation and cardiovascular reflex response (127). Additionally, EA and MA have different efficacy for IS. The efficacy of EA is better than the efficacy of MA for recovering nerve defects, improving quality of life (128), and strengthening limb motor function (129).

Acupoints are associated with the specificity of the acupuncture effect, and acupoint application is one of the factors that influence efficacy. MA at Neiguan (PC6) for 60 s was more effective than that at 5 s and 180 s in MCAO rats (130). The EA at Hanyan (GB4), Xuanlu (GB5), Xuanli (GB6), and Qubin (GB7) showed more significant recovery than at Baihui (GV20) and Shuigou (GV26) (103). Another study showed that acupuncture at Neiguan (PC6) was more effective than acupuncture at Chize (LU5) and Shuigou (GV26) (131). Zhang et al. used cluster analysis to illustrate that the Neiguan (PC6), Weizhong (BL40), Chize (LU5), Sanyinjiao (SP6), and Shuigou (GV26) groups had the most valid and suitable acupuncture parameters (132). Acupoint selection in the current study was unsystematic, because little attention was paid for the accurate stimulation of neurons. More research is needed to explore the most effective acupoints to accurately stimulate the corresponding neural signaling pathways.

Overall, different types of acupuncture treatment, duration, operation time, retention, output frequency, and acupoint selection will affect the effect of acupuncture treatment.

## 5 Prospect and future direction

Immune responses are complicated cascade reactions after an IS. However, current studies about immune organs, cells, and cytokine responses remain shallow. The immune mechanisms of IS are appealing and deserve to be explored. For example, myeloid cells that take on a foamy appearance are a key driver of the inflammatory response after an IS. Targeting lipid accumulation in foam cells may be a promising strategy for accelerating recovery from IS (133).

A phase III trial indicated that the tumor necrosis factor receptor-immunoglobulin G1, which controls a single inflammatory cytokine, was ineffective in the incidence or resolution of organ dysfunction (134). Programmed cell death protein 1 (PD-1) inhibitors, which are immune checkpoint inhibitors, can regulate cellular immunity in patients with cancer. However, PD-1 inhibitors cause endocrine autoimmune diseases, gastrointestinal discomfort, hepatotoxicity, rashes, and thyroid dysfunction at the same time (135). The inflammatory cascade reaction after ischemia is produced by multiple cytokines and not by a single cytokine; therefore, successful treatment of IS requires inhibition of multiple cytokines. Acupuncture improves disease by modulating the release of multiple biomolecules into the microenvironment, where they can activate the neuroendocrine–immune network to achieve holistic modulation (136). Therefore, acupuncture is a promising potential treatment for IS through the regulation of the immune network. The underlying mechanisms of acupuncture in IS require further exploration.

The vagus nerve and hypothalamic–pituitary–adrenal (HPA) axis are important components of neuroimmune interactions because they control immune function and inflammatory responses. The hypothesis of acupuncture neuroimmune regulation in the IS model by eliciting the vagal–adrenal and spinal–sympathetic axes has been raised. Ma et al. confirmed the neuroanatomy of EA at ST36 with low intensity driving the vagal–adrenal axis to produce anti-inflammatory effects (137), while EA at ST25 with high intensity activates NPY splenic noradrenergic neurons via the spinal–sympathetic axis (138). In particular, the current intensity is critical. EA at ST36 improved inflammatory arthritis in Lyme disease-susceptible C3H mice by stimulating the sciatic–vagal nerve (139). Since current studies on the neuroimmune regulation mechanism in acupuncture treatment for IS have not focused on the vagus nerve, HPA axis, or splenic sympathetic nerve, they will be a promising target.

Natural killer (NK) cells are cytotoxic lymphocytes of the innate immune system. Acupuncture also regulates NK cells by modulating the expression of the NK cell receptor CD94, protein tyrosine kinase, adhesion molecule vascular cell adhesion molecule-1 (VCAM-1), and protein tyrosine phosphatase (140). There has been no research on the mechanism of acupuncture that focuses on the modulation of NK cells. Therefore, NK cells are a promising target for exploration.

Immunoregulators can be used to treat IS, offering a new therapeutic approach to IS treatment. Acupuncture aims to restore and maintain the dynamic equilibrium of the immune system instead of strengthening or inhibiting the immune system. These evidences show that acupuncture is a potential adjunct to immune regulation in IS, which has not been previously proposed in published reviews.

## 6 Conclusion

Acupuncture treatment is an ancient non-drug treatment, and it shows fewer side effects and beneficial therapeutic effects. This review summarizes the progress in understanding the immunomodulatory mechanisms of acupuncture treatment for IS and provides a theoretical framework for further mechanism research. In summary, the above evidences suggest that acupuncture has the potential to modulate the immune response induced by IS, which is related to its neuroprotective effects. The exploration of the mechanisms of acupuncture promotes its global impact.
