# Corrigendum: The immunomodulatory mechanism of acupuncture treatment for ischemic stroke: research progress, prospects, and future direction

**DOI:** 10.3389/fimmu.2024.1468179

**Published:** 2024-08-16

**Authors:** Hongjun Kuang, Xinzhou Zhu, Huan Chen, Han Tang, Hong Zhao

**Affiliations:** ^1^ Department of Acupuncture and Moxibustion, Shanghai University of Traditional Chinese Medicine, Shenzhen Hospital, Shenzhen, China; ^2^ Department of Acupuncture and Moxibustion, Luohu District Hospital of Traditional Chinese Medicine, Shenzhen, China; ^3^ The Brain Cognition and Brain Disease Institute (BCBDI), Shenzhen Institute of Advanced Technology, Chinese Academy of Sciences, Shenzhen, China; ^4^ Shenzhen-Hong Kong Institute of Brain Science-Shenzhen Fundamental Research Institutions, Shenzhen, China; ^5^ Institute of Acupuncture and Moxibustion, China Academy of Chinese Medical Science, Beijing, China

**Keywords:** immunomodulatory mechanism, acupuncture treatment, ischemic stroke, research progress, future direction, prospects

In the published article, there was an error in affiliation 1. Instead of “1 Department of Acupuncture and Moxibustion, Shenzhen Luohu Hospital of Traditional Chinese Medicine (Shenzhen Hospital of Shanghai University of Traditional Chinese Medicine), Shenzhen, China”, it should be split into two separate affiliations “1 Department of Acupuncture and Moxibustion, Shanghai University of Traditional Chinese Medicine, Shenzhen Hospital, Shenzhen, China” and “2 Department of Acupuncture and Moxibustion, Luohu District Hospital of Traditional Chinese Medicine, Shenzhen, China”. Besides, the affiliation of Hong Zhao should only be “2 Department of Acupuncture and Moxibustion, Luohu District Hospital of Traditional Chinese Medicine, Shenzhen, China”.

The authors apologize for this error and state that this does not change the scientific conclusions of the article in any way. The original article has been updated.

